# Transcriptomic Profiling of Ca^2+^ Transport Systems during the Formation of the Cerebral Cortex in Mice

**DOI:** 10.3390/cells9081800

**Published:** 2020-07-29

**Authors:** Alexandre Bouron

**Affiliations:** Genetics and Chemogenomics Lab, Université Grenoble Alpes, CNRS, CEA, INSERM, Bâtiment C3, 17 rue des Martyrs, 38054 Grenoble, France; alexandre.bouron@cea.fr

**Keywords:** calcium channels, Na/Ca exchanger, calcium pumps, endoplasmic reticulum, calcium stores, cerebral cortex, neurogenesis

## Abstract

Cytosolic calcium (Ca^2+^) transients control key neural processes, including neurogenesis, migration, the polarization and growth of neurons, and the establishment and maintenance of synaptic connections. They are thus involved in the development and formation of the neural system. In this study, a publicly available whole transcriptome sequencing (RNA-Seq) dataset was used to examine the expression of genes coding for putative plasma membrane and organellar Ca^2+^-transporting proteins (channels, pumps, exchangers, and transporters) during the formation of the cerebral cortex in mice. Four ages were considered: embryonic days 11 (E11), 13 (E13), and 17 (E17), and post-natal day 1 (PN1). This transcriptomic profiling was also combined with live-cell Ca^2+^ imaging recordings to assess the presence of functional Ca^2+^ transport systems in E13 neurons. The most important Ca^2+^ routes of the cortical wall at the onset of corticogenesis (E11–E13) were TACAN, GluK5, nAChR β2, Cav3.1, Orai3, transient receptor potential cation channel subfamily M member 7 (TRPM7) non-mitochondrial Na^+^/Ca^2+^ exchanger 2 (NCX2), and the connexins CX43/CX45/CX37. Hence, transient receptor potential cation channel mucolipin subfamily member 1 (TRPML1), transmembrane protein 165 (TMEM165), and Ca^2+^ “leak” channels are prominent intracellular Ca^2+^ pathways. The Ca^2+^ pumps sarco/endoplasmic reticulum Ca^2+^ ATPase 2 (SERCA2) and plasma membrane Ca^2+^ ATPase 1 (PMCA1) control the resting basal Ca^2+^ levels. At the end of neurogenesis (E17 and onward), a more numerous and diverse population of Ca^2+^ uptake systems was observed. In addition to the actors listed above, prominent Ca^2+^-conducting systems of the cortical wall emerged, including acid-sensing ion channel 1 (ASIC1), Orai2, P2X2, and GluN1. Altogether, this study provides a detailed view of the pattern of expression of the main actors participating in the import, export, and release of Ca^2+^. This work can serve as a framework for further functional and mechanistic studies on Ca^2+^ signaling during cerebral cortex formation.

## 1. Introduction

The divalent cation calcium (Ca^2+^) is a universal intracellular signaling messenger [1]. The cytosolic concentration of free ionized Ca^2+^ ([Ca^2+^]_i_) in quiescent cells is estimated to be in the 10–100 nM range. However, various pathophysiological conditions elevate [Ca^2+^]_i_, which, in turn, influences a myriad of biological processes, such as gene expression, neurogenesis, neuronal migration, axon growth, synaptic transmission, synaptic plasticity, and the maintenance of synaptic networks [1,2,3,4]. The duration, magnitude, and spatiotemporal characteristics (subcellular localization and frequency) of the cytosolic Ca^2+^ rise are crucial parameters controlling the Ca^2+^-dependent intracellular signaling cascades. Schematically, [Ca^2+^]_i_ changes reflect an entry of Ca^2+^ from the extracellular milieu or a release from internal stores. These two processes are often interrelated and, thus, cooperate to influence [Ca^2+^]_i_.

An influx of Ca^2+^ is mainly mediated by Ca^2+^-conducting channels. These channels constitute a rather complex and diverse family, comprising structurally, pharmacologically, and functionally different proteins with distinct patterns of expression and subcellular localization. [Ca^2+^]_i_ rises are counterbalanced by systems that maintain low [Ca^2+^]_i_ levels. This is achieved via the extrusion, storage, and/or intracellular accumulation of Ca^2+^ into compartments. Therefore, at the cellular level, a vast number of proteins (e.g., channels, pumps, exchangers, and Ca^2+^-binding proteins) are involved in the handling of Ca^2+^ and the shaping of its cytosolic variations. Any insult modifying the abundance, distribution, and activity of these proteins is likely to affect cell functions and survival.

In the mammalian brain, the cerebral cortex is composed of six superimposed cellular layers formed during embryogenesis. In mice, this formation takes place between embryonic days 10–11 (E10–E11) and E17–E18 [5,6] and requires coordinated and regulated sequences of cell proliferation, migration, morphological differentiation, and synaptogenesis [7,8,9]. Any alteration of these key stages can ultimately alter the growth and development of the cerebral cortex. Understanding the formation of this brain area is of crucial importance as a variety of developmental neuropathologies and neurological disorders are associated with cortical abnormalities [8,10]. As changes in [Ca^2+^]_i_ levels play pivotal roles during the formation of the cortex [2,3,11], it is important to characterize the repertoire of actors participating in the transport of Ca^2+^ through membranes.

The aim of this report was to provide a detailed picture of the expression of genes encoding the putative plasma membrane and organellar Ca^2+^-transporting proteins during embryonic cortical formation and development. The work is based on a recent genome-wide transcriptome sequencing (RNA-Seq) analysis estimating gene expression [12]. Tissues were collected at four ages: embryonic days 11 (E11), 13 (E13), and 17 (E17), and post-natal day 1 (PN1) corresponding to four crucial stages: when the neural progenitor cells begin to differentiate into neurons (E11), the peak (E13) and end of neurogenesis (E17), followed by the beginning of the maturation process and circuit assembly (PN1). The report is divided into two parts: the first part is a genome-scale profile of gene expression that is further subdivided into five chapters covering the following topics: (1) channels, (2) Ca^2+^ ATPases, (3) exchangers, (4) antiporters, and (5) mitochondrial actors. The second part of the report is devoted to the main functional Ca^2+^ transport systems present in E13 cortical cells.

## 2. Materials and Methods

### 2.1. Animals

The experiments were conducted in accordance with the French legislation and the European Community Council Directive of 24 November 1986 (86/609/EEC). C57Bl6/J mice (Charles River, l’Arbresle, France) were housed in the same room under a 12 h light/12 h dark cycle with ad libitum access to food and water. They were exposed to an enriched environment in agreement with the Animal Welfare Committee of the Commissariat à l’énergie atomique et aux énergies alternatives (CEA) Grenoble. To enable the dating of the embryos, we crossed the animals at night once a week and then males were removed before 9 a.m. This day was counted as E0.

### 2.2. Primary Cultures of Cortical Cells

Primary cultures of embryonic cortical neurons were prepared from E13 C57BL6/J mice as previously described [13,14,15], according to an experimental protocol approved by the ethical committee of the CEA’s Life Sciences Division (CETEA) (#A14-006).

### 2.3. Single-Cell Calcium Imaging

These experiments were conducted with the Ca^2+^ probe Fluo4 as previously described [13,16,17]. Briefly, cells were loaded with 5 µM Fluo4/AM for 25 min, washed, and kept for another 5–10 min in a standard recording saline solution containing (in mM) 150 NaCl, 5 KCl, 1 MgCl_2_, 2 CaCl_2_, 5.5 glucose, and 10 4-(2-hydroxyethyl)-1-piperazineethanesulfonic acid (HEPES) (pH 7.4). All these procedures were performed at room temperature and in the dark. Coverslips were then mounted on the stage of an inverted Axio Observer A1 microscope with a Fluar 40× oil immersion objective lens (1.3 NA) (Carl Zeiss, Marly le Roi, France) and a charge-coupled device camera (CoolSnap HQ2, Princeton Instruments, Roper Scientific, Evry, France). The experimental setup, equipped with a DG-4 wavelength switcher (Princeton Instruments, Roper Scientific, France), was driven by MetaFluor (Universal Imaging, Roper Scientific, Evry, France). The cytosolic Ca^2+^ signals were acquired from cells bodies at a sampling rate of 0.2 Hz.

### 2.4. Single-Cell Zinc Imaging

Cytosolic zinc (Zn^2+^) rises were monitored following the same experimental conditions and setup as described above, except the cells were incubated with the fluorescent Zn^2+^ dye FluoZin3 [15,18]. When used, the Ca^2+^ and Mg^2+^ free recording solution consisted of (in mM): 150 NaCl, 5 KCl, 5.5 glucose, and 10 HEPES (pH 7.4). The Fluo4 and FluoZin-3 signals were expressed as F/F0, with F being the fluorescence at each time point and F0 being the mean baseline fluorescence monitored for 1 min before the addition of any substance. The coverslips were used once and never reused.

## 3. Results and Discussion

### 3.1. Transcriptomic Analysis

The results are expressed in transcripts per million (TPM) [12]. The complete dataset is accessible on the GEO repository at the following address: https://www.ncbi.nlm.nih.gov/geo/query/acc.cgi?acc=GSE154677.

#### 3.1.1. Channels

##### Voltage-Gated Ca^2+^ Channels (VGCC)

**Figure 1:**
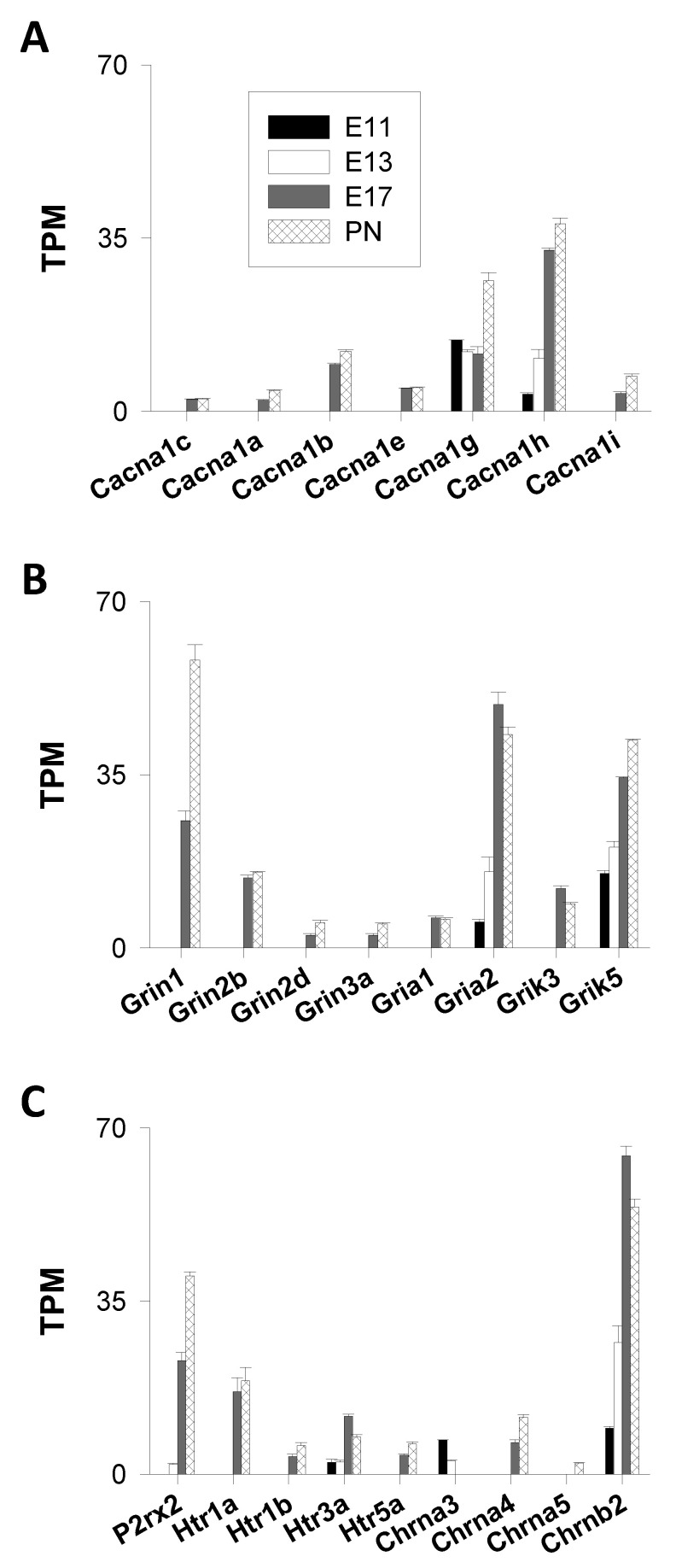
Genes coding for voltage-gated and ligand-gated Ca^2+^ channels (VGCC and LGCC, respectively). The figure shows the expression of genes coding for the pore-forming subunits of VGCC (**A**); ionotropic glutamate receptor subunits (**B**); and adenosine triphosphate (ATP)/purinergic, 5-hydroxytryptamine (serotonin), and nicotinic acetylcholine receptor subunits (**C**). Throughout this study, the abundance of transcripts is presented in transcripts per million (TPM), and only genes displaying TPM values ≥ 2 were considered to be significantly expressed. Four ages were studied: embryonic days 11 (E11), 13 (E13), and 17 (E17), and post-natal day 1 (PN1). The mean +/− standard error of mean (SEM) is shown from *n* = 3 distinct biological samples. All the data presented were extracted from a genome-wide transcriptome sequencing (RNA-Seq) analysis (Hasna et al., 2019).

Ten genes (*Cacna1s, Cacna1c, Cacna1d, Cacna1f, Cacna1a, Cacna1b, Cacna1e, Cacna1g, Cacna1h,* and *Cacna1i*) encoding the pore-forming subunits Ca_v1.1-1.4_, Ca_v2.1-2.3_, and Ca_v3.1-3.3_ were analyzed. Transcripts of the following three genes coding for L-type VGCC were not detected in this RNA-Seq analysis: *Cacna1s*, *Cacna1d,* and *Cacna1f*. They had TPM values < 2 at each time point. Five transcripts were not detected at the beginning of corticogenesis (E11 and E13); however, their abundance increased thereafter: *Cacna1c* (Ca_v1.2_, L-type), *Cacna1a* (Ca_v2.1_, P/Q-type), *Cacna1b* (Ca_v2.2_, N-type), *Cacna1e* (Ca_v2.3_, R-type), and *Cacna1i* (Ca_v3.3_, T-type) (Figure 1A). On the other hand, two genes (*Cacna1g* and *Cacna1h*) were expressed at all time points. They code for Ca_v3.1_ (*Cacna1g*) and Ca_v3.2_ (*Cacna1h*), two low-threshold T-type Ca^2+^ channels. These two genes were the most expressed VGCC genes with TPM values ranging from ≈10 to ≈35 (Figure 1A). At E11 and E13, *Cacna1g* mRNA (Ca_v3.1_) was the major VGCC transcript, whereas *Cacna1h* mRNA (Ca_v3.2_) predominated at E17 and PN1, showing a clear shift in the expression pattern of genes encoding T-type VGCC. *Cacna1h* was the most strongly induced gene with a number of transcripts increasing by a factor ≈10 from E11 to PN1. The third most expressed gene of this group was *Cacna1b* (Ca_v2.2_, N-type) with TPM values of ≈10. The other VGCC genes displayed much lower TPM values (<5) (Figure 1A).

Overall, cells of the immature cortical wall were found to be equipped with distinct types of VGCC even at E11 and E13. Their mRNA levels increased with a clear augmentation noted at E17 compared to E13. Western blot experiments showed the presence of Ca_v3.1_ proteins in the E14 murine forebrain [19]. Electrophysiological recordings provided evidence for the presence of functional T-type VGCC in cells of the ventricular zone at E15 [20], and in E13 and E15 cortical neurons in primary culture [21,22]. An entry of Ca^2+^ via functional VGCC was also observed at E14 in the ventricular zone [23]. Altogether, these data point to the expression of functional T-type VGCC at very early stages of the cortical development [19,20]. They were the predominant VGCC transcripts throughout corticogenesis, from E11 to PN1, indicating that Ca^2+^ influx through low-threshold VGCC plays important roles during the formation of the rodent cortex.

##### Ligand-Gated Cation Channels (LGCC)

The following cationic ionotropic neurotransmitter receptors were studied: glutamate, ATP/purinergic, nicotinic acetylcholine, and 5-hydroxytryptamine (serotonin) receptors.

1. Ionotropic Glutamatergic Receptors

A total of 18 genes were selected (*Gria*, *Grin*, *Grik*, and *Grid*) covering the four classes of ionotropic glutamate receptors α-amino-3-hydroxy-5-methyl-4-isoxazolepropionic acid (AMPA) (or GluA1-4), *N*-methyl-d-aspartate (NMDA) (or GluN1-3), kainate (or GluK1-5), and delta (or GluD) receptors, respectively [24]. No transcripts of the *Grid1–2* genes (coding for ionotropic glutamate receptors delta 1 and 2) were found.

(a) AMPA Receptors (GluA1-4)

Among the four genes studied (*Gria1–4*), two had TPM < 2 (*Gria3, Gria4*) and one gene (*Gria1*) was detected only at the end of corticogenesis (E17 and PN1), whereas *Gria2* (GluA2) was expressed at all ages tested. The number of *Gria2* transcripts increased by a factor ≈10 between E11 and the end of corticogenesis (E17), indicating a strong regulation in the expression of this AMPA receptor gene (Figure 1B).

(b) NMDA Receptors (GluN1–3)

The distinct types of NMDA receptor subunits (GluN1, GluN2A–2D, and Glu3A–3B) are encoded by seven genes. Transcripts of three of them (*Grin2a*, *Grin2c*, and *Grin3b*) could not be detected (TPM values < 2). Transcripts of the other four genes (*Grin1*, *Grin2b*, *Grin2d,* and *Grin3a*) could not be appreciated before E17. The predominantly expressed NMDA receptor gene was *Grin1*. There was a marked enhancement of the *Grin1* expression between E17 and PN1, with TPM values increasing from 25 to 58 (around a twofold change) (Figure 1B). *Grin1* encodes GluN1, an NMDA subunit widely distributed in the brain [25].

(c) Kainate Receptors (GluK1-5)

*Grik5* and, to a lesser extent, *Grik3* were the predominant genes of this subgroup. They encode GluK5 and GluK3, high and low affinity kainate receptor subunits, respectively. *Grik5* was the only kainate receptor gene to be expressed throughout corticogenesis. GluK5 forms functional receptors when associated with other kainate receptor subunits, such as GluK1-3. When expressed alone, they are mainly found in the endoplasmic reticulum and are not targeted to the cell surface [26]. Together with *Gria2*, *Grik5* was the main ionotropic glutamatergic subunit gene expressed at the onset of corticogenesis. *Grik5* expression was strongly enhanced with TPM values increasing from 15 to 42 (around a threefold change) (Figure 1B).

2. Ionotropic Purinergic Receptors

Seven genes coding for ionotropic purinergic receptors (P2XR) were considered: *P2rx1*–7. The RNAseq analysis indicated that *P2rx2* (encoding P2X2 receptors) was the sole gene of this subgroup expressed during corticogenesis and that *P2rx2* transcripts were not detected at E11 (with TPM < 2). The TPM values were 2, 23, and 40 at E13, E17, and PN1 (Figure 1B). Expression of the *P2rx2* gene was considerably enhanced between E13 and E17 (with a ≈10-fold change in the TPM values), indicating an important role during the formation of the cortical layers. The abundance of transcripts were augmented by a factor of two between E17 and PN1. Overall, this represented a 20-fold increase in the *P2rx2* mRNA abundance between the peak of corticogenesis (E13) and the postnatal period. The *P2rx2* gene was one of the most upregulated genes of this study.

3. Ionotropic Serotoninergic (5-HT) Receptors

Only one ionotropic 5-HT receptor gene was expressed throughout corticogenesis: *Htr3a*. The TPM values of *Htr3a* increased from ≈2–3 at E11–E13 to ≈12 at E17 before declining to ≈7 at PN1. Three other transcripts were detected but only starting at E17 (the end of corticogenesis) and onward: *Htr1a*, *Htr1b,* and *Htr5a*. With TPM values of 16–18 (at E17 and PN1), the *Htr1a* gene predominated over the other members (*Htr1b* and *Htr5a*) that displayed TPM values of 3–6 at the same ages (Figure 1C). 5-HT3 was the sole type of ionotropic serotoninergic receptor present at the early stages of corticogenesis, whereas 5-HT1A predominated at the end of corticogenesis. As for VGCC, there was a shift in the expression pattern of genes encoding 5-HT receptors.

4. Nicotinic Acetylcholine Receptors

Concerning nicotinic acetylcholine receptors (nAChR), 16 genes were considered: *Chrna1–10* (nAChR alpha 1–10 subunits); *Chrnb1–Chrnb4* (nAChR beta 1–4 subunits); and *Chrnd, Chrne,* and *Chrng* (nAChR delta, epsilon, and gamma subunits, respectively). Transcripts of only three genes were detected: *Chrna3* (nAChR α3), *Chrna4* (nAChR α4), and *Chrnb2* (nAChR β2). Overall, *Chrnb2* was, by far, the most expressed gene of this subfamily of cation channels. *Chrnb2* was expressed at all ages. The abundance of *Chrnb2* transcripts exhibited a sevenfold increase throughout corticogenesis with TPM values of 9, 27, 64, and 54 at E11, E13, E17, and PN1, respectively (Figure 1C). The other two transcripts, *Chrna3* (nAChR α3) and *Chrna4* (nAChR α4), were found at much lower levels (TPM values around 7–3 and 6–11 for *Chrna3* and *Chrna4*, respectively). These two genes were oppositely regulated—βon the one hand, *Chrna3* was expressed at the onset of corticogenesis; on the other hand, the expression of the *Chrna4* gene began at the end of corticogenesis (E17). Both observations were in agreement with previous findings [27]. In summary, this transcriptomic analysis indicated that (α3, β2) and (α4, β2) were the predominant nicotinic subunits of the cortical wall at the beginning (E11–E13) and the end of neurogenesis (E17–PN1), respectively.

When analyzing the genes encoding cationic ionotropic neurotransmitter receptor subunits at the four time points (E11, E13, E17, and PN1) (Figure 1B,C), there appeared to be an overall rise in the TPM values—starting at 2–15 TPM at the beginning of corticogenesis (E11), and reaching values of 15–65 TPM at the end of corticogenesis (E17 and PN1). This reflects an increase in the abundance of transcripts throughout corticogenesis. The most highly expressed genes were kainate *Grik5* (GluK5), nicotinic cholinergic *Chrnb2* (nAChR β2), and AMPA *Gria2* (GluA2). At the end of neurogenesis (E17–PN1), the major transcripts of this subgroup were *Grin1* (GluN1), *Grin2b* (GluN2B), and *P2rx2* (P2X2 receptors). Of note, among the five most expressed genes at the end of corticogenesis (E17 and PN1) and encoding cationic ionotropic neurotransmitter receptor subunits, two of them (*Grin1* and *Grin2b*) encoded NMDA subunits (GluN1 and GluN2B).

This study was focused on the expression of the genes encoding Ca^2+^-permeable channels. In this context, it is important to recall that the mRNA of the AMPA receptor GluA2 undergoes a post-transcriptional editing process giving rise to functionally distinct channels deriving from the same gene (*Gria2*)—in its edited form, GluA2 forms Ca^2+^-impermeable channels. In the absence of edited GluA2 subunits, multimeric GluA2 receptors are Ca^2+^-conducting channels [28,29]. The non-edited form (giving rise to Ca^2+^-permeable GluA2 subunit) is detectable during embryogenesis only, found, for instance, in neuronal progenitor cells where the post-transcriptional regulation process of the GluA2 mRNA could be involved in the induction of neurogenesis [30]. However, even in the immature brain, the great majority (>99%) of the GluA2 mRNA is considered to be in the edited form, implying that most AMPA receptors are Ca^2+^-impermeable [28]. Therefore, the Ca^2+^ permeability of AMPA channels is merely dictated by the absence or presence of GluA2 subunits.

##### Transient Receptor Potential (TRP) Channels

Mammalian TRP channels form a large superfamily of cation channels that is divided into six subfamilies: TRPA (ankyrin), TRPC (canonical), TRPM (melastatin), TRPML (mucolipin), TRPP (polycystin), and TRPV (vanilloid) [31,32]. The expression of 28 TRP channel genes was analyzed. Apart from TRPV5 and TRPV6, two highly Ca^2+^-selective permeable TRP channels, most TRP channels are non-selective cation channels that are permeable to Na^+^ and Ca^2+^ ions, with the exception of TRPM4 and TRPM5, which are Ca^2+^-impermeable [33].

1. TRPA Channels

This subgroup of TRP channels is restricted to one member—TRPA1, but no transcript of the *Trpa1* gene could be detected.

2. TRPC Channels

**Figure 2:**
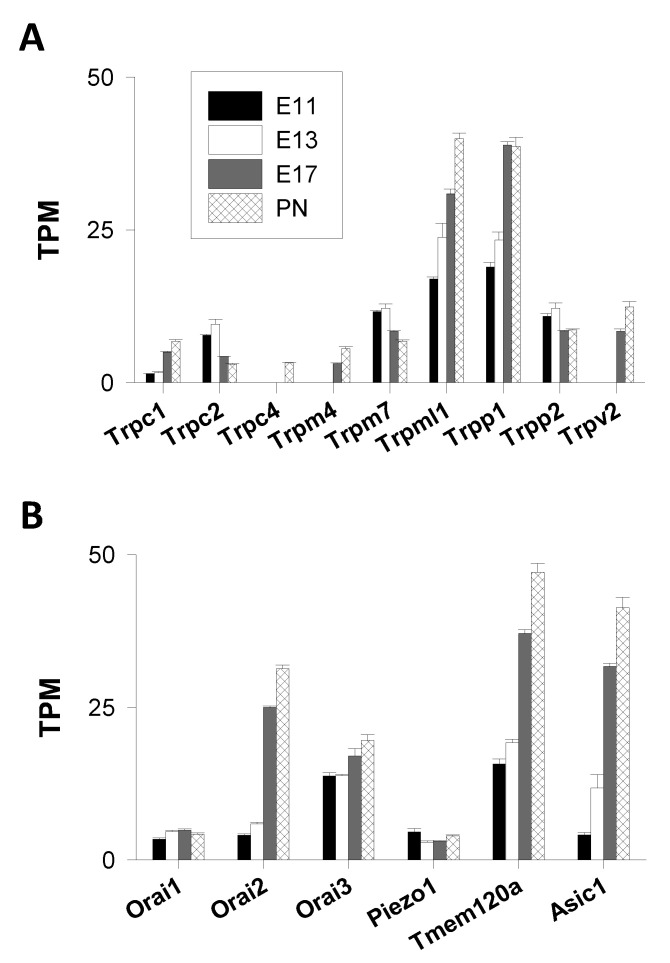
Genes coding for transient receptor potential (TRP), Orai, Piezo, TMEM120, and acid-sensing ion channel (ASIC) channels. The figure shows the expression of genes coding for TRP channels (**A**), and Orai, Piezo, TMEM120A, and ASIC1 channels (**B**). The mean +/− standard error of mean (SEM).

Three *Trpc* genes (*Trpc1*, *Trpc2*, and *Trpc4)* out of seven were found to be expressed but at very low levels (TPM values < 10). Transcripts of *Trpc1* and *Trpc4* were present at the end of corticogenesis, whereas the *Trpc2* gene was expressed throughout cortical development. However, the *Trpc2* mRNA abundance peaked at E13 and then declined at later stages, indicating a repression of the expression of the *Trpc2* gene (Figure 2A).

3. TRPM Channels

Two *Trpm* genes (out of eight) were expressed: *Trpm7* and *Trpm4*. The latter codes for a Ca^2+^-impermeable channel [33] and was thus excluded from the analysis. The expression of the *Trpm7* gene was repressed during corticogenesis with TPM values declining from ≈11 at E11 to ≈6 at PN1 (Figure 2A).

4. TRPML Channels

High levels of *Trpml1* mRNAs were observed, and their abundance was augmented throughout corticogenesis (from 17 to 40 TPM) (Figure 2A). With TPM values < 2, the other *Trpml* genes (*Trpml2–3*) did not appear to be expressed.

5. TRPP Channels

The expression of the following *Trpp* genes was analyzed: *Trpp1* (Pkd1, polycystin 1 or PC1), *Trpp2* (Pkd2 or polycystin 2), *Trpp3* (PKD2L1), and *Trpp5* (PKD2L2). Only two *Trpp* genes had TPM values > 2: *Trpp1* and -2. They exhibited distinct regulation patterns—*Trpp1* expression was induced, whereas *Trpp2* expression was slightly repressed during corticogenesis (Figure 2A). For all time points considered, *Trpp1* mRNAs predominated over *Trpp2* mRNAs. The *Trpp1* gene (Pkd1) encodes a plasma membrane protein (named TRPP1 or PC1) that is not regarded as an ion channel per se [34]. TRPP1 is rather a component of a receptor–ion channel complex comprising TRPP2, a non-selective Ca^2+^-conducting cation channel [35].

6. TRPV Channels

*Trpv2* was the only *Trpv* gene expressed, but not before E17, indicating an induction of the gene expression at late stages of corticogenesis (Figure 2A). The TPM values of *Trpv2* were comparable to *Trpm7* and *Trpp2* (around 10 TPM). As no other *Trpv* transcripts were found, TRPV2 is likely to form homomultimeric channels in the cortex, which is in agreement with previous data [36].

Only four *Trp* channel genes displayed TPM values > 2 at the beginning of corticogenesis (E11–E13): *Trpml1*, *Trpp2, Trpm7,* and *Trpc2*. The other *Trp* members had TPM values < 2, a finding consistent with the notion that most *Trp* channel transcripts are present at low levels in many organs [37]. Transcripts of *Trpc1* and *Trpv2* could be quantified at the end of corticogenesis (E17) whereas *Trpc4* transcripts were noticeable only postnatally. Previous studies addressed the question of the expression of murine *Trp* channel genes but focused on a specific subset of genes, without considering, for instance, the TRPP and TRPML channel subfamilies [37,38]. Here, all mammalian TRP subfamilies were analyzed. This provides a clearer picture of the *Trp* gene expression profile during murine corticogenesis.

TRPML1 is an endo/lysosomal channel ubiquitously expressed and permeable to cations. This channel plays crucial roles in intracellular trafficking and membrane fusion/fission. Mutations in the *Trpml1* gene are associated with a childhood neurodegenerative lysosomal storage disorder known as mucolipidosis type IV [39,40]. *Trpml1* is the major *Trp* gene expressed in the murine cortex with TPM values two to three times larger than those of the other TRP members. Its gene expression is positively regulated from E11 to PN1.

Like TRPML1, TRPP2 (Pkd2) is also an intracellular non-selective Ca^2+^-permeable channel but is primarily located in the endoplasmic reticulum (ER). Found in primary cilia, TRPP2 appears to play a critical role in organ morphogenesis, required for the left–right body axis formation [35]. TRPP2 has been described as a mechanosensitive channel, and mutations in the human *Trpp2* gene are associated with an autosomal dominant polycystic kidney disease. However, its roles in brain cells are unclear. This study uncovered that *Trpml1* and *Trpp2* were the two most highly expressed *Trp* channel genes. In addition, *Trpm7* was also one of the most prominent *Trp* genes (Figure 2A), which is in agreement with previous reports [37,38].

*Trpv* transcripts appeared at later stages, with *Trpv2* being one of the most expressed *Trp* genes at the end of corticogenesis (E17 and PN1). Several functions were ascribed to TRPV2, including noxious heat sensing and osmo- and chemo-sensation. However, the physiological roles, as well as the mechanism of activation, of native TRPV2 channels in the brain are still largely unknown. TRPV2 is expressed by astrocytes, and Ca^2+^ entry through TRPV2 was proposed as playing a role in neurite outgrowth [36].

Concerning *Trpc*, genes were expressed either at early stages (E11 and 13) or postnatally (at PN1), with a shift in the gene expression pattern—*Trpc2* predominated early on, whereas *Trpc1* was the predominant *Trpc* member at later stages. Ca^2+^ entry through TRPC channels was proposed as participating in various aspects of neuronal development, such as neuronal survival or synaptogenesis [41]. TRPC2 was originally described as a diacylglycerol-activated channel that is highly expressed in vomeronasal sensory neurons, playing a role in pheromone detection in mice [42]. Unexpectedly, *Trpc2* was identified as the most expressed *Trpc* gene at the onset of corticogenesis, with a marked repression of its expression after E17. Whether TRPC2 channels are involved in corticogenesis is unknown. TRPC1, the founding member of the TRPC subfamily, has a broad tissue expression. TRPC1 appears to form heteromultimeric complexes comprising other TRP proteins (TRPC4/5, TRPV4/6, and TRPP1) or stromal interaction molecule 1(STIM1). However, studies aiming at understanding its role in cellular Ca^2+^ signaling have raised much debate and therefore the functions of TRPC1 proteins remain elusive. The expression of the *Trpc1* gene was induced at the end of corticogenesis.

##### Orai Channels

Orai are highly selective plasma membrane Ca^2+^ channels [43,44]. *Orai3* was the major *Orai* gene at E11 with mRNA levels that remained nearly constant during corticogenesis. The *Orai2* gene was the most upregulated *Orai* gene (the TPM values increased by a factor ≈8 between E11 and PN1), as well as the predominantly expressed *Orai* gene at the end of corticogenesis and postnatally (with ≈30 TPM at PN1). This result is consistent with the view that Orai2 is a major Orai channel in cells of the neural system, at least in the cerebral cortex [45,46]. Conversely, *Orai1*, with TPM values <5, had the weakest mRNA level at each time point (Figure 2B). The expression of *Orai1* was not developmentally regulated.

##### Stretch-Activated Channels

This family comprises two members: Piezo1 and -2 [47]. Only the *Piezo1* gene was expressed with TPM values of 3–4 throughout corticogenesis, indicating no developmental regulation in its expression (Figure 2B). Mechanosensitive Piezo1 channels were shown to play a role in axon growth in the developing brain [48]. TACAN (or Tmem120A) is a recently identified mechanosensitive ion channel permeable to Ca^2+^ [49]. It is highly expressed in the sensory neurons of the dorsal root ganglia but RNA-Seq analysis showed that it was also present in the immature cerebral cortex. The expression of the gene was strongly upregulated—the number of TPM increased from 14 to 37 between E11 and E17, a 2.6-fold increase in transcript abundance (Figure 2B). The exact contribution of TACAN in Ca^2+^ signaling is unknown; however, the high expression of the *Tmem120A* gene indicates that mechanical forces, via mechanosensitive channels, could play a role in the growth, migration, and/or differentiation of cortical cells. 

##### Acid-Sensing Ion Channels (ASIC)

Channels of the ASIC family are proton-gated channels. Six members are known in rodents (ASIC1–6). They form Na^+^- and K^+^-conducting ion channels, apart from homomeric ASIC1a, which displays a low permeability for Ca^2+^ [50]. Interestingly, the *Asic1* gene was the only *Asic* gene expressed during corticogenesis. A 10-fold increase in its TPM values was noted, increasing from 4 (at E11) to 41 TPM (at PN1) (Figure 2B). Therefore, the expression of this gene was strongly upregulated. The other *Asic* genes were not expressed (TPM values < 2), except for *Asic4*, which was detected at PN1 only (with TPM values of 3), indicating that the expression of this gene was induced postnatally (not shown). ASIC1 channels are present in central nervous system neurons with high levels of ASIC1 proteins in the hippocampal and cortical regions of mice brains [51]. Activation of ASIC1 causes an intracellular Ca^2+^ rise in cultured embryonic cortical neurons [52]. ASIC1 participates in the central treatment of noxious messages [51]. This protein is involved in synaptic plasticity as its loss impairs hippocampal long-term potentiation and learning tasks [53,54]. In vitro data indicated that ASIC1-dependent currents were developmentally regulated during the maturation of cortical neurons, with a decrease in their Ca^2+^ permeability [52].

The following two chapters (I.1.7 and I.1.8) cover the expression of genes encoding intracellular Ca^2+^-conducting channels.

##### Inositol 1,4,5-Trisphosphate and Ryanodine Receptors (IP_3_R, RyR), and Two-Pore Channels (TPC)

**Figure 3:**
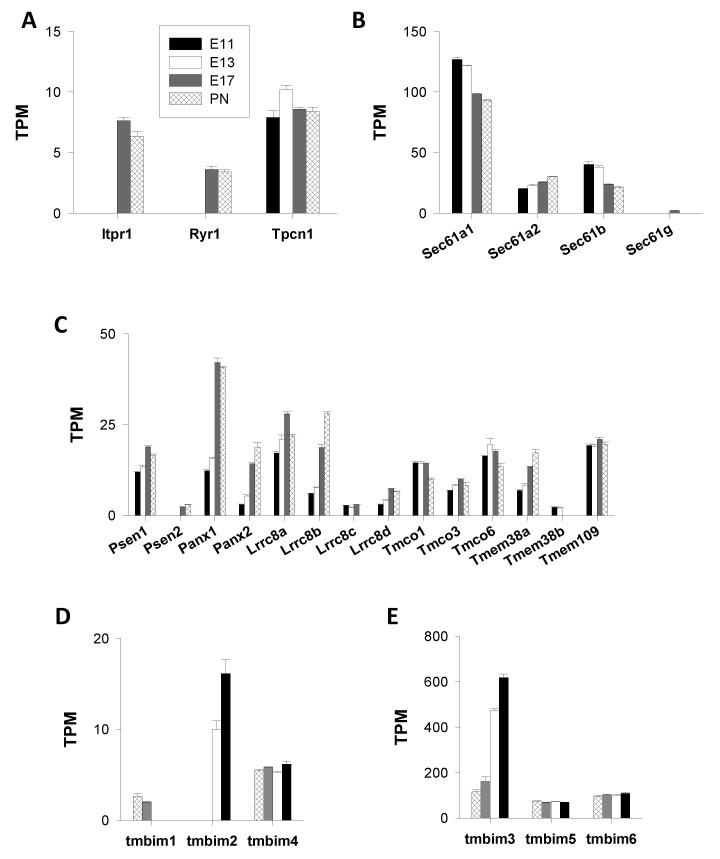
Genes coding for inositol 1,4,5-trisphosphate and ryanodine receptors (IP3R and RyR, respectively), and two-pore channels (TPC) and leak channels. In this figure the expression of genes coding for IP3 and ryanodine receptors, and two-pore channels (**A**) and the putative leak channels (see text) (**B**–**E**) are shown. For the sake of clarity, the results were plotted on different graphs due to the large range of transcripts per million (TPM) values (from <10 to >500 TPM).

The genes *Itpr1–3, Ryr1–2,* and *Tpcn1–2* code for inositol 1,4,5-trisphosphate receptors (IP_3_R1–3), ryanodine receptors (RyR1–2), and two-pore channels (TPC), respectively. These proteins constitute three important families of intracellular Ca^2+^ channels that are mainly found in the ER (IP_3_R and RyR) and endo/lysosomes (TPC). The RNA-Seq data showed that *Tpcn1* was the most expressed gene, with a peak in its mRNA abundance at E13 (Figure 3A). No other gene of this subgroup was expressed at E11–E13. TPCs are principally found in the membrane of acidic compartments, such as endo-lysosomes where they appear to function as regulators of vesicle fusion [55]. The expression of *Itpr1–3* and *Ryr1–2* was increased at later stages, notably *Itpr1* (IP_3_R1) and (RyR1) with TPM values of ≈8 and ≈4 at PN1, respectively (Figure 3A). Of note, genes coding for the organellar Ca^2+^ channels IP_3_R, RyR, and TPC had TPM values far below those of the plasmalemmal Ca^2+^ channel genes described in the previous paragraphs.

##### Intracellular “leak Channels”

The pharmacological blockade of sarco/endoplasmic reticulum Ca^2+^ ATPases (SERCA) (see below, paragraph I.2 “Ca^2+^ ATPases”) initiates a passive release of Ca^2+^ mediated by so-called “*leak channels*” that appear to be molecularly distinct from the classical ER Ca^2+^ channels (e.g., IP_3_R and RyR) (but see [56]). Thus far, many putative candidates have been postulated, including translocons, pannexins, leucine-rich repeat-containing 8 (LRRC8), mitsugumin 23 (MG23), presenilin [57], TRPC1 [58], Orai3 [59], and members of the Bax inhibitor-1 motif containing (TMBIM) protein family [60]. Here, the following gene transcripts were considered:

1. Translocons

Translocons are protein complexes forming pores that allow the translocation of polypeptides across membranes. The functional core of mammalian translocons involves Sec61 proteins [61]. These can mediate ER Ca^2+^ release, making Ca^2+^ leak channels ubiquitous, particularly Sec61A1 [62,63]. The following *Sec61* transcripts were analyzed: *Sec61a1–a2, Sec61b,* and *Sec61g*. *Sec61a1* was the major *Sec61* gene with a high abundance of transcripts (TPM values >90). Interestingly, the expression of *Sec61a1* was repressed with TPM values diminishing from 127 (at E11) to 93 TPM (at PN1). A similar trend was observed for the less-expressed gene *Sec61b*, with TPM values declining from 40 to 22 (Figure 3B). Conversely, *Sec61a2*, although less expressed, was positively regulated (from 20 to 30 TPM). The expression of *Sec61g* was detected only at PN1 (Figure 3B). For the sake of clarity, the analysis of the other putative leak channel genes is shown in a distinct graph because the TPM values were much smaller, ranging from 2 to 40 (see Figure 3C).

2. Presenilins

Presenilins are ER proteins that are suggested to contribute to ER Ca^2+^ leak channels [64]. This view has, however, been challenged by [65], who found no evidence for a role of presenilins in ER Ca^2+^ leak channels. The primarily expressed presenilin gene was *Psen1* (Figure 3C). The abundance of transcripts in the cortical wall raised by ≈50% during corticogenesis (from ≈12 TPM at E11 to ≈19 TPM at E17). The presenilin 2 gene (*Psen2*) was weakly expressed, and *Psen2* transcripts were detected only at E17 and PN1 (Figure 3C).

3. Pannexins

The family of pannexin channels comprises three members (Panx1-3) [66,67]. Two genes (*Panx1* and *Panx2*) were expressed and the abundance of the transcripts increased throughout corticogenesis, with *Panx1* being the predominant pannexin gene at all ages. The expression of *Panx1* was upregulated after E13, where the TPM values increased from 15 (at E13) to 42 (at PN1), which corresponds to a ≈3.5-fold increase (Figure 3C). The expression of the minor *Panx2* gene was further stimulated as the TPM values increased from 3 to 18, reflecting an approximate sixfold augmentation.

4. LRRC8

Pannexins have significant sequence similarity with leucine-rich repeat-containing 8 (LRRC8) proteins. Among the five LRRC8 proteins known thus far, one of them (LRRC8B) behaves as a Ca^2+^ leak channel [68]. The analysis of the abundance of the transcripts of the five *Lrrc8* genes (*Lrrca* to *Lrrce*) indicated that *Lrrc8a* was the most expressed *Lrrc* gene throughout corticogenesis; however, *Lrrc8b*, displaying higher TPM values at E17 and PN1, exhibited strong developmental regulation as its mRNA abundance increased approximately fivefold between E11 (≈6 TPM) and PN1 (≈28 TPM) (Figure 3C). This regulation pattern displayed some similarities with *Panx2*. Collectively, *Lrrc8b* transcripts were less abundant than *Panx1* transcripts.

5. TMCO1

The transmembrane and coiled-coil domains 1 (*Tmco1*) gene encodes a channel (TMCO1) preventing ER Ca^2+^ overload [69]. In the *Tmco* gene family, three members dominated: *Tmco6*, *Tmco1,* and *Tmco3* (Figure 3C). Their levels of transcripts varied weakly during corticogenesis. The other *Tmco* genes did not appear to be expressed.

6. TRIC

Two subtypes of trimeric intracellular cation channels (TRIC) are known: TRIC-A and -B, deriving from distinct genes (*Tmem38a–b*). Recent studies identified TRICA–B proteins as central actors controlling the release of Ca^2+^ out of the ER [57,70]. Figure 3C shows that *Tmem38a* was the most highly expressed *Tmem38* gene throughout corticogenesis with TPM values increasing from ≈7 to 17. Transcripts of the minor *Tmem38b* gene were found principally at the beginning of corticogenesis (E11–E13) and then declined (Figure 3C). TRIC-A has been shown to co-cluster with STIM1/Orai1 proteins and functions as negative modulator of store-operated Ca channels and oscillatory Ca^2+^ signals [71].

7. Mitsugumin23

This protein, localized in intracellular membranes, was proposed to participate in the ER Ca^2+^ release [72]. Transcripts of the gene (also named transmembrane protein 109 or *Tmem109*) were found at all time points with TPM values of 19–20; however, no developmental regulation was noticed (Figure 3C).

8. Bax Inhibitor-1 Motif-Containing (TMBIM) Proteins

Members of the Bax inhibitor-1 motif-containing (TMBIM) protein family are important regulators of intracellular Ca^2+^ homeostasis. They have been suggested to form Ca^2+^ leak channels of the ER [60,73]. The expression of the six known genes (*Tmbim1*–6) is given in Figure 3D,E. *Tmbim3* and *Tmbim5–6* were the most expressed genes; however, they displayed distinct expression patterns—*Tmbim5–6* expression was constant whereas *Tmbim3* was strongly upregulated with a marked increase at the end of corticogenesis (E17).

Overall, *Tmbim3* and *Tmbim5–6* were, far behind *Sec61a1*, the most strongly expressed genes coding for putative intracellular Ca^2+^ leak channels.

#### 3.1.2. Ca^2+^ ATPases

**Figure 4:**
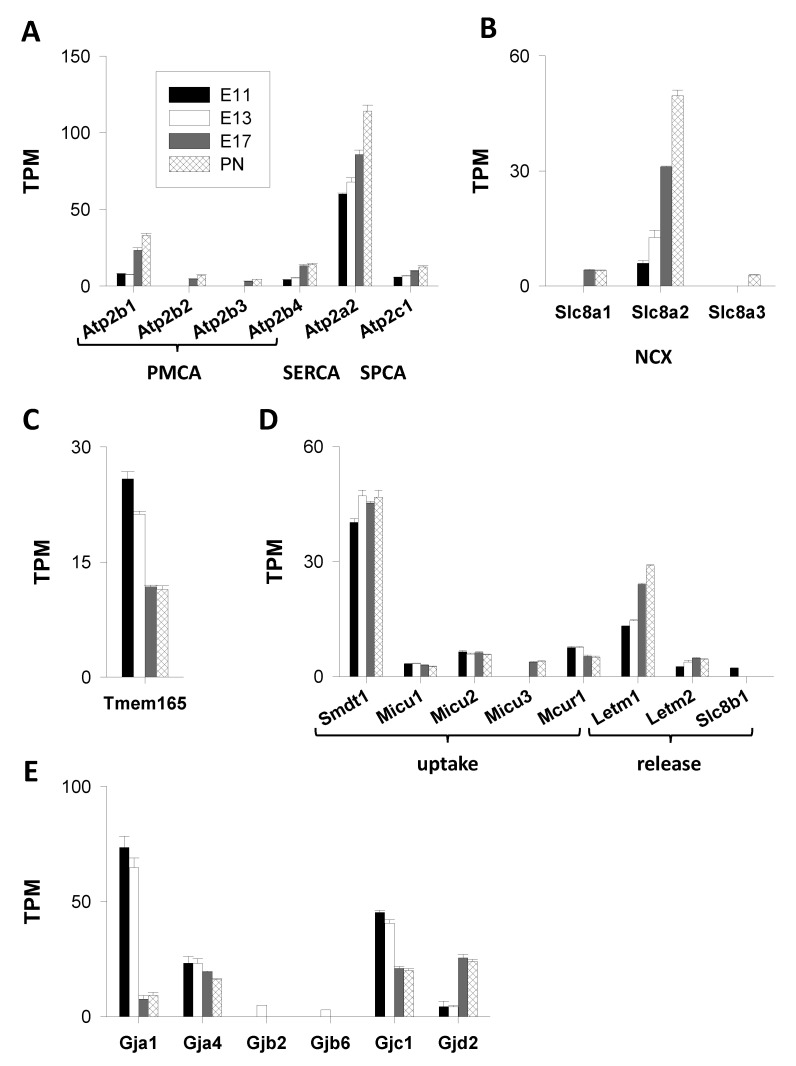
Genes coding for Ca^2+^ pumps, exchangers, antiporters, mitochondrial actors, and connexins. Genes coding for plasma membrane Ca^2+^ ATPase (PMCA), sarco/endoplasmic reticulum Ca^2+^ ATPases (SERCA), and secretory pathway Ca^2+^-ATPase (SPCA) are presented in panel (**A**). Panel (**B**) shows the expression of genes coding for non-mitochondrial Na^+^/Ca^2+^ exchangers (NCX). Panels (**C**,**D**) present the Ca^2+^/H^+^ antiporter TMEM165 and mitochondrial actors, respectively. Panel (**E**) gives an overview of the genes coding for connexins.

The group of Ca^2+^ ATPases encompasses diverse types of pumps with distinct subcellular localizations and pharmacological properties [74]. Plasma membrane Ca^2+^ ATPases (PMCA) are encoded by four genes (*Atp2b1–4*). The RNA-seq data showed that *Atp2b1* (encoding PMCA1) was the major PMCA gene, followed by *Atp2b4* (PMCA4). Transcripts of *Atp2b1* and *Atp2b4* (PMCA4) were detected at each age and their abundance increased during development, particularly at E17. The expression of these two PMCA genes was thus positively regulated at later stages of cortical formation. This was also the case of the minor *Atp2b2* and *Atp2b3* genes encoding PMCA2 and PMCA3, whose expression was not detected at early ages (E11–E13) (Figure 4A).

Intracellular Ca^2+^ pumps are derived from two gene families: *Atp2c* (*Atp2c1–c2*) and *Atp2a* (*Atp2a1–a3*), encoding Ca^2+^ ATPases of the Golgi network (secretory pathway Ca^2+^-ATPase or SPCA1–2) and of the sarco/endoplasmic reticulum (SERCA1–3), respectively [74]. The *Atp2a2* gene, encoding SERCA2, was by far the main actor. In addition to SERCA genes, only one gene encoding SPCA was expressed—*Atp2c1*. This mRNA expression profile was comparable to that of *Atp2b4* (PMCA4). A general feature of these Ca^2+^ ATPase genes (PMCA, SPCA, and SERCA) is the enhancement of their expression throughout corticogenesis. The most induced gene was *Atp2b1* (PMCA1) with mRNA levels increasing by a factor of around four from E11 to PN1 (Figure 4A).

#### 3.1.3. Exchangers

The *Slc8a1-a3* and *Slc24a1-a5* genes encode the non-mitochondrial Na^+^/Ca^2+^ (NCX) and Na^+^/Ca^2+^/K^+^ (NCKX) exchangers. As shown in Figure 4B, only *Slc8a* genes were expressed, with *Slc8a2*, coding for NCX2, being by far the major *Ncx* gene. Its expression increased steadily from ≈6 to ≈50 TPM, revealing a greater than eightfold rise in transcript abundance between E11 and PN1. At E19, virtually all cortical neurons displayed a functional NCX mechanism but not glial progenitors [75]. This finding likely explains the strong induction of the *Slc8a2* gene after E13 where the neurons outnumbered the progenitor cells. It has been proposed that, during cortical development, NCX activity may play an important role in Ca^2+^ homeostasis of differentiating neurons but not in glial progenitor cells [75]. The *Slc8a1* and *Slc8a3* genes were much less expressed, with transcripts detected solely at the end of corticogenesis (Figure 4B).

#### 3.1.4. Cation Antiporter

Although detected in endosomes, lysosomes, and at the cell surface, the transmembrane protein 165 (TMEM165) is described as a Golgi-localized protein and thought to function as a putative cation (Ca^2+^/H^+^) antiporter. Recent data demonstrated that TMEM165 can transport ions, such as Ca^2+^ and Mn^2+^, that are required for protein glycosylation [76,77]. Transcripts of the *Tmem165* gene were found at all time points (Figure 4C). They were more abundant at the onset of neuro-corticogenesis with TPM values of >20 at E11–E13, declining thereafter to 11 at E17–PN1. This indicates that, at embryonic ages corresponding to important sequences of cell division and neurogenesis (such as E11–E13), which are associated with important phases of synthesis and post-translation modifications (such as glycosylation) of proteins, there is a substantial need for Ca^2+^ in the Golgi apparatus.

#### 3.1.5. Mitochondrial Actors (Figure 4D)

This section covers the actors participating in mitochondrial Ca^2+^ uptake and release.

##### Mitochondrial Ca^2+^ Uptake

The mitochondrial Ca^2+^ uniporter (MCU) is regarded as the primary entry route for Ca^2+^. MCU is a multi-molecular complex of the inner mitochondrial membrane that is highly selective for Ca^2+^. The core components consist of MCU, mitochondrial calcium uniporter dominant negative beta subunit (MCUb), and Emre (an essential MCU regulator), with MCU and MCUb being the pore-forming subunits. No transcripts of the *Mcu* and *Mcub* genes were detected (TPM < 2) but single-pass membrane protein with aspartate rich tail 1 *Smdt1*, the gene encoding for the non-pore-forming subunit Emre (important for the assembly and function of the MCU complex [78]), was highly expressed (with TPM values of 40–50) (Figure 4D). The *Micu1–3* genes, encoding MICU1–3, three key regulators of the MCU complex [78], were, however, weakly expressed (TPM values of 5–6). *Micu2* was the major *Micu* gene. Although its roles in MCU complex assembly and function remain unclear, MCUR1 appears to play a role in mitochondrial Ca^2+^ homeostasis [78]. Its gene (*Mcur1*) was also weakly expressed with TPM values of approximately 5–7. The expression of these mitochondrial genes was not developmentally regulated (Figure 4D).

##### Mitochondrial Ca^2+^ Release

Extrusion of Ca^2+^ from the mitochondrial matrix appeared to be achieved by the Na^+^/Ca^2+^/Li^+^ exchanger (NCLX), encoded by the *Slc8b1* gene, distinct from the plasma membrane Na^+^/Ca^2+^ exchanger. Transcripts of this *Slc8b1* gene were found exclusively at E11 (2 TPM) but not at later stages. The mitochondrial exchanger NCLX is thought to participate in Ca signaling. The failure to detect transcripts may be due to detection problems. Although this issue remains disputed [79], leucine zipper EF hand-containing transmembrane protein 1 (LETM1), a protein coded by the *Letm1* gene, was identified as a putative mitochondrial H^+^/Ca^2+^ exchanger [80] that could extrude Ca^2+^ out of the mitochondria [81]. The expression of the *Letm* genes, *Letm1* and *Letm2,* was analyzed. The abundance of transcripts increased after E13 and peaked at later ages. Overall, *Letm1* was the primary gene, with TPM values increasing twofold between E13 and PN1 (from 13 to 29 TPM, respectively), pointing to a clear regulation of this gene during corticogenesis (Figure 4D). LETM1 maintains the mitochondrial osmotic balance [82]; however, its role in mitochondrial Ca^2+^ dynamics is not firmly established.

#### 3.1.6. Connexins

Alongside channels, pumps, exchangers, and transporters, gap junctions formed by connexins participate in Ca^2+^ movements through membranes. They are, for instance, found in neuroblasts of the rodent ventricular zone, allowing the coupling between adjacent cells within a cluster [83]. In the embryonic brain, gap junctions are required for the occurrence of spontaneous cytosolic fluctuations [84]. The expression of the connexin genes is reported in Figure 4E. Out of the 21 connexin genes [85], only four were expressed at all ages (*Gja1*, *Gja4*, *Gjc1*, and *Gjd2*). They code for gap junction proteins alpha-1 (connexin 43 or CX43), alpha-4 (CX37), gamma-1 (CX45), and delta 2 (CX36), respectively. *Gja1*, the major connexin gene, was highly expressed at the beginning of corticogenesis (with TPM values of ≈75 at E11). As with *Gja4* and *Gjc1*, *Gja1* expression was repressed particularly after E13. TPM values decreased nearly eightfold between E11 and PN1.

The *Gjd2* gene displayed a completely different expression pattern—weakly expressed at E11–E13, it became the major gene of this family at the end of corticogenesis (E17–PN1). The TPM values increased by a factor of six between E11 and PN1 (from 4 to 24 TPM) (Figure 4E). In addition, transcripts of two genes were detected at one age only (E13): *Gjb2* and *Gjb6* (gap junction proteins beta-2 and -6, CX26 and CX30, respectively). They had low TPM values (≈2–4). In terms of copy numbers, the *Gja1* gene was expressed at a much higher level than any other plasma membrane actor, such as *Cacna1g*, *Grik5,* or *Chrnb2*. This highlights the key roles played by these gap junction-dependent Ca^2+^ signals during the early phases of cortical development [84,86]. These gap junctions are prominent structures of the ventricular zone at the beginning of neurogenesis. Hence, neurons uncouple from clusters of coupled cells while migrating out of the ventricular zone [83].

#### 3.1.7. Expression Profile of The Main Ca^2+^ Transport Systems Throughout Corticogenesis

The 10 most abundant transcripts at each age (E11, E13, E17, and PN1) were examined. Table 1 and Table 2 give an overview of the top 10 genes coding for plasma membrane (Table 1) and intracellular (Table 2) Ca^2+^ transport systems. This provides an overview of the temporal pattern of the expression of the major genes.

### 3.2. Presence of Functional Ca^2+^ Transport Systems at E13

The second part of this study focuses on the functional Ca^2+^ transport systems of E13 cortical cells. The different layers of the cortical wall are formed sequentially, following an inside-out order. Therefore, the first post-mitotic neurons generated reside in the deepest cortical layers and fulfil important roles during corticogenesis. Any Ca^2+^ dyshomeostasis at the early stages of neurogenesis is likely to influence the growth and formation of the neocortex, which, in turn, may affect the cognitive, motor, and sensory functions.

#### 3.2.1. Functional Ca^2+^ Transport Systems of The Plasma Membrane

A list of the 10 most expressed genes encoding plasma membrane Ca^2+^ channels is given in Table 1. The presence of functional channels generating intracellular Ca^2+^ changes was tested by single-cell fluorescent Ca^2+^ imaging with the fluorescent probe Fluo-4. This study focused its attention on *Chrnb2* (AchR β2), *Grik5* (GluK5), *Orai3* (Orai3), and *Trpm7* (TRPM7). The contribution of the connexin genes and TACAN was not addressed. E13 cortical neurons express NCX2 proteins and display a functional Na^+^/Ca^2+^ exchanger [87]. Therefore, the *Slc8a2* gene product was also not covered.

##### Acetylcholine Nicotinic Receptors AChR β2

In this set of experiments, the recording saline contained atropine (10 µM) to block muscarinic acetylcholine receptors. The application of nicotine (10 µM) generated a cytosolic Ca^2+^ elevation in only 1 out of 172 cells tested from two distinct batches of cells (not shown). This suggests that E13 cortical cells lack functional Ca^2+^-conducting acetylcholine nicotinic receptors.

##### Orai3

**Figure 5:**
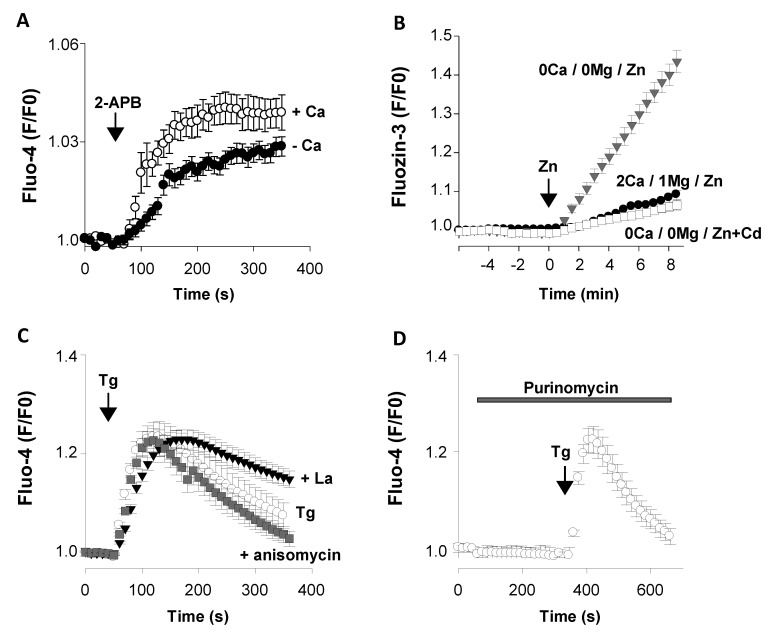
Presence of functional Ca^2+^ channels at E13. Live-cell Ca^2+^ and Zn^2+^ imaging experiments were conducted to assess the presence of functional Ca^2+^-transport systems in dissociated E13 cortical cells loaded with Fluo-4 (**A**,**C**,**D**) or FluoZin-3 (**B**). The baseline Fluo-4 (or FluoZin-3) fluorescence was recorded for 1 min and averaged (F0). Changes in the Fluo-4 (or FluoZin-3) fluorescence (F) over time were expressed as F/F_0_. Mean +/− SEM. When not visible, the error bars are smaller than the symbols. Figure 5A shows Fluo-4 signals recorded following the application of 50 µM 2-APB to primary E13 cortical neurons maintained in a nominally Ca^2+^-free saline (filled circles, *n* = 77 cells/5 dishes) and in a 2 mM Ca^2+^-containing saline (open circles, *n* = 66 cells/4 dishes). For the sake of clarity, only one point of three is shown. Figure 5B, the presence of functional TRPM7 channels was assessed using the fluorescent Zn^2+^ probe FluoZin-3. The recording saline contained 2 mM Ca^2+^ and 1 mM Mg^2+^ and was supplemented with Zn acetate (100 µM) when indicated (arrow, time 0) (filled circles, *n* = 112 cells/6 dishes). In additional experiments, the recording saline was switched to a nominally Ca^2+^- and Mg^2+^-free saline 4 min prior to the addition of zinc acetate (time 0) without (gray triangles, *n* = 167 cells/7 dishes) or with 5 µM GdCl_3_ (open squares, *n* = 101 cells/5 dishes). One point of four is shown. As displayed in Figure 5C, in these experiments, a nominally Ca^2+^-free saline was used to rule out the contribution of external sources of Ca^2+^. Thapsigargin (Tg, 200 nM), a blocker of SERCA, was applied when indicated (vertical arrow) either alone (open circles, *n* = 117 cells/6 dishes) or together with 100 µM LaCl_3_ (filled triangles, *n* = 81 cells/4 dishes). The protein synthesis inhibitor anisomycin (500 µM for 1 h/37 °C) did not affect the Tg-evoked Ca^2+^ signal (gray squares, *n* = 109 cells/5 dishes). One point of three is shown. Figure 5D shows how puromycin (250 µM) was applied to cells kept in a nominally Ca^2+^-free saline (horizontal gray bar). Tg was also added (arrow) to ensure that cells had replete Ca^2+^ stores (*n* = 89 cells/5 dishes); one point of three is shown

Orai3 channel activity can be enhanced by 2-aminoethyl diphenyl borate (2-APB, 50 µM) independently of STIM1-2 and of the filling status of the ER Ca^2+^ stores [88,89]. As illustrated in Figure 5A, the application of 50 µM 2-APB to primary E13 cortical neurons provoked a small but consistent cytosolic Ca^2+^ rise (open circles) that was significantly reduced upon the removal of external Ca^2+^ (filled circles; Figure 5A). This showed that 2-APB stimulated a release as well as an entry of Ca^2+^ in E13 cortical cells. Orai3 was detected at the cell surface and intracellularly in the membrane of the ER, mediating a 2-APB-trigerred Ca^2+^ release. This Orai3-dependent Ca^2+^ route is distinct from the thapsigargin-evoked Ca^2+^ leak pathway [59]. In aggregate, E13 cortical cells expressed 2-APB-sensitive channels located at the cell surface and intracellularly, generating Ca^2+^ signals of small amplitudes. At the mRNA level, all types of Orai channels were found in the cortical wall at E13. E13 cortical cells also display a functional store-operated Ca entry (SOCE) pathway [15,90].

##### TRPM7

Although TRPM7 can be found intracellularly [91], it is assumed to function primarily as a plasma membrane conductance sensitive to external cations. In neurons, lowering the extracellular concentrations of Ca^2+^ and Mg^2+^ activates TRPM7 channels that are highly permeable to Zn^2+^ ions [92,93]. This property was exploited to examine the presence of functional TRPM7 channels at E13. To that aim, cortical cells were loaded with the fluorescent Zn^2+^ probe FluoZin-3 [18]. The cells were first maintained in a saline containing 2 mM Ca^2+^ and 1 mM Mg^2+^ before introducing 100 µM Zn acetate into the recording saline (time 0, arrow). This was associated with a small elevation of the FluoZin-3 fluorescence (Figure 5B, filled circles). In another set of experiments, the cells were bathed for 4 min in a nominally Ca^2+^- and Mg^2+^-free saline to enhance TRPM7 activity [93] prior to the addition of zinc acetate (at time 0). Removing the Ca^2+^ and Mg^2+^ ions had no effect on the basal FluoZin-3 fluorescence. However, the external application of 100 µM Zn acetate caused a robust and sustained elevation of the FluoZin-3 fluorescence (Figure 5B, gray triangles). This response, observed in all cells tested (167 cells from 7 dishes), was strongly depressed by a low concentration of the cation channel blocker gadolinium (5 µM GdCl_3_) (Figure 5B, open squares). Altogether, E13 cortical cells expressed functional TRPM7-like channels sensitive to the removal of extracellular Ca^2+^ and Mg^2+^ ions and permeable to Zn^2+^ [93,94,95].

#### 3.2.2. Functional Intracellular Ca^2+^ Transport Systems

A list of the 10 most expressed E13 genes involved in the transport of Ca^2+^ through intracellular membranes is given in Table 2. In this section, two genes were considered: *Atp2a2* (SERCA2) and *Sec61a1* (translocon).

##### The Ca^2+^ Pump SERCA2

A*tp2a2* was the most expressed SERCA gene throughout corticogenesis (Figure 4A). The presence of functional SERCA2 pumps was assessed with the specific inhibitor thapsigargin (Tg) [96]. In cells with replete ER Ca stores, the application of Tg was followed by a [Ca^2+^]_i_ rise, revealing a passive leakage of Ca^2+^ out of Tg-sensitive Ca^2+^ compartments.

To exclude the contribution of external Ca^2+^, the following experiments were performed with a nominally Ca^2+^-free saline. At E13, Tg elevated [Ca^2+^]_i_ in a majority of cells (117 cells out of 130 cells tested, 90%) (Figure 5C, open circles) [14,15,90]. This Tg-induced Ca^2+^ signal showed that E13 cells possessed a functional Ca^2+^ storage system able to deliver Ca^2+^ into Tg-sensitive stores. The transient elevation of [Ca^2+^]_i_ provoked by Tg reflected the presence of efficient Ca^2+^ homeostatic mechanisms able to restore low cytosolic levels of Ca^2+^. The *Atp2b1* gene encodes PMCA1, which pumps Ca^2+^ out of cells. The activity of this Ca^2+^ ATPase is blocked by the trivalent cation lanthanum (La^3+^) [97,98]. When added together with Tg, La^3+^ (0.1 µM) delayed the decaying phase to resting basal Ca^2+^ levels (Figure 5C, filled triangles). This La^3+^-induced impairment of the extrusion of Ca^2+^ indicated that E13 cortical neurons express functional PMCA1. When compared to PMCA, SERCA appeared as the major Ca^2+^ ATPase at E13. A*tp2a2* (SERCA) and *Atp2b1* (PMCA) transcripts were found at high and low levels, respectively, indicating that E13 cells possessed a very efficient ER Ca^2+^ storage system in comparison to the extrusion route via Ca^2+^ pumps.

##### The Leak Channel Translocon

The *Sec61a1* gene was one of the most expressed genes coding for putative Ca^2+^ leak channels. The contribution of *Sec61a1* to the passive release of Ca^2+^ was investigated using the protein synthesis inhibitor puromycin that opens the translocon. For instance, puromycin (200 µM) depletes the Ca^2+^ stores of the ER in vascular smooth muscle cells [99]. However, when tested on E13 cortical cells, 250 µM puromycin did not affect the resting basal [Ca^2+^]_i_ levels (Figure 5D). It also did not abolish the Tg-evoked Ca^2+^ rise. This showed that the ER Ca^2+^ stores were unaffected by puromycin (Figure 5D). Anisomycin, another inhibitor of the protein synthesis that closes the translocon, had no effect on the Tg-evoked Ca^2+^ release (Figure 5C, gray squares). As indicated in Chapter I.1.8, many candidates have been postulated to contribute to the ER Ca^2+^ leak, such as translocons, pannexins, LRRC8, MG23, presenilin, TRPC1, Orai3, and members of the Bax inhibitor-1 motif-containing (TMBIM) protein family. Our data indicated that the protein encoded by the *Sec61a1* gene was not expressed or did not contribute to the Ca^2+^ leak pathway, as has been reported by others [99,100].

### 3.3. Summary

**Figure 6:**
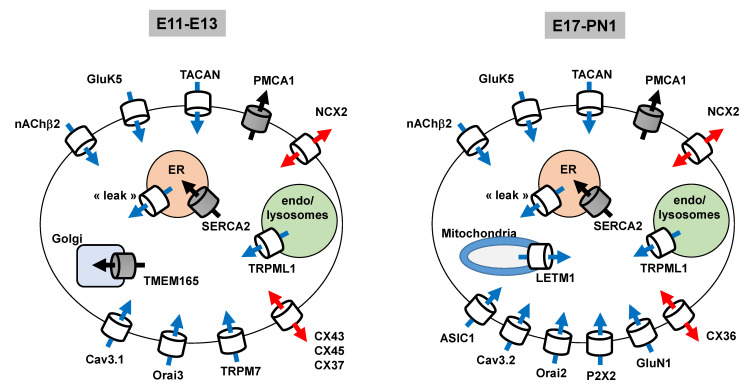
Ca^2+^ transport system during corticogenesis. Graphical summary of the key results of this RNA-Seq analysis. The data are derived from Table 1 and Table 2, uncovering the list of the 10 most expressed genes at a given age. The scheme shows the gene products (and their putative subcellular localization) of the most expressed genes and their differential expression pattern between the beginning (E11–E13) and the end of neurogenesis (E17–PN1). On top are the Ca^2+^ transport systems deriving from genes coding for cell surface proteins that are highly expressed throughout corticogenesis. At the bottom are the cell surface Ca^2+^ transport systems coded by genes displaying a high expression profile at the onset of corticogenesis (E11–E13) (left panel) and end of corticogenesis (E17PN1) (right panel). The expression profile of the most prominent genes coding for internal Ca^2+^ transport systems is also shown. Blue and black arrows: actors contributing to elevate and decrease [Ca^2+^]i, respectively. Red arrows: actors permitting a bidirectional transport (uptake/export) of Ca^2+^, or between adjacent cells. Collectively this gives an overview of the most abundant transcripts found in the cortical wall during neurogenesis.

Figure 6 summarizes some of the main results of this study. The scheme presents the putative subcellular localization of the products of the 10 most expressed genes and their expression pattern between the beginning (E13–E17) and end of corticogenesis (E17–PN1). At the transcriptomic level, the most important Ca^2+^ uptake routes of the cell surface at the onset of corticogenesis (Figure 6) are channels formed by TACAN, GluK5, Orai3, Cav_3.1_, TRPM7, and nAChR β2. These are mechano-sensitive (TACAN), ligand-gated (GluK5 and nAChR β2), voltage-gated (Cav3.1), and Mg/ATP-regulated constitutively activated channels (TRPM7). Orai3 has been described as a store-operated Ca channel; however, it also appears to be a component of arachidonic acid-regulated calcium-selective (ARC) channels.

In addition, the connexins CX43/CX45/CX37 allow an intercellular coupling, whereas NCX2, depending on its reversal potential, can work in a forward (Ca^2+^ export) or backward (Ca^2+^ uptake) mode. These actors (CX and NCX) permit a bidirectional transport of Ca^2+^ through the plasma membrane (or between adjacent cells). Intracellularly, TRPML1 and the uncharacterized “leak” channels form the two prominent Ca^2+^ release pathways at all ages. In front of these numerous routes that contribute to elevating cytosolic Ca^2+^ levels, SERCA2 and PMCA1 appeared as the crucial systems to bring Ca^2+^ back to its resting basal levels. TMEM165 is another actor able to store Ca^2+^ in internal compartments; however, this contribution is less prominent at the end of corticogenesis. A more numerous and diverse population of Ca^2+^ uptake systems was observed in the cortical wall at the end of neurogenesis (E17 and onward). In addition to the actors listed above, certain prominent Ca^2+^-conducting systems emerged, including ASIC1, Orai2, P2X2, and GluN1. 

## 4. Conclusions

The major purpose of this study was to perform a transcriptional profiling to survey the global expression patterns of genes coding for Ca^2+^ transport systems of the cortical wall during mouse corticogenesis. This permitted the establishment of the developmental expression profiles of the major genes involved in Ca^2+^ transport through membranes. In the second part of the report, certain genes were selected and live-cell Ca^2+^ (or Zn^2+^) imaging experiments were performed to verify the presence of functional proteins at E13. Overall, numerous Ca^2+^ transporter genes were expressed in the embryonic cerebral cortex. Collectively, there is a large diversity of Ca^2+^ transport systems at the cell surface that contrasts with the paucity of prominent internal Ca^2+^ transport systems.

One limitation of this work is the possibility that some transcripts were missed due to the existence of genes with unknown functions. New ion channels are continuously being uncovered. For instance, it was recently shown that the stretch-activated nonselective cation channel TMEM63B is an osmosensor that can mediate the Ca^2+^ influx [101]. This example indicates that it cannot be excluded that genes coding for Ca^2+^-transport proteins and contributing to Ca^2+^ signaling were missed in the present work. Another limitation of this work is the fact that many, if not all, ion channels require dedicated and specific auxiliary subunits that are of the upmost physiological importance for their assembly/maturation/trafficking/surface expression and/or ion transport activities and properties. Although crucial for the biogenesis and appropriate activity of ion channels, the mRNA expression of these regulatory subunits was not investigated.

It is also important to consider that some actors selected in this report may function as pathways for cations distinct from Ca^2+^. For example, TRPM7 is a Ca^2+^-conducting pathway but it may function as a physiological Mg^2+^ transport system [102]. In addition, two other limitations must be introduced: only certain transcripts are translated to proteins, and this transcriptomic approach did not consider the post-translational modifications known to influence the activity and/or subcellular localization of proteins. Altogether, this study provided a detailed view of the pattern of expression of the main actors participating in the import, export, and release of Ca^2+^ during the formation and development of the cerebral cortex. This study can serve as a valuable resource and provide a framework for further functional and mechanistic studies on Ca^2+^ signaling during cerebral cortex formation.

## Figures and Tables

**Table 1:** List of the top 10 genes coding for plasma membrane Ca^2+^ transport systems. Ranking is also given./means not present in the top 10 list at a given age. The analysis encompasses a total of 17 genes.

Ca Transport System	*Gene*	Protein	Rank Number			
			E11	E13	E17	PN
Connexins	*Gja1*	CX43	1	1	/	/
Connexins	*Gjc1*	CX45	2	2	/	/
Connexins	*Gja4*	CX37	3	4	/	/
Voltage-gated channels	*Cacna1g*	Cav_3.1_	6	10	/	/
Other channels	*Trpm7*	TRPM7	8	9	/	/
Store-operated channels	*Orai3*	Orai3	7	7	/	/
Exchanger	*Slc8a2*	NCX2	/	8	6	3
Connexins	*Gjd2*	CX36	/	/	8	/
Voltage-gated channels	*Cacna1h*	Cav_3.2_	/	/	4	8
Ligand-gated channels	*Grin1*	GluN1	/	/	7	1
Ligand-gated channels	*P2rx2*	P2X2	/	/	/	7
Store-operated channels	*Orai2*	Orai2	/	/	9	10
Acid-sensing channels	*Asic1*	ASIC1	/	/	5	6
Ligand-gated channels	*Chrnb2*	nAChR β2	10	3	1	2
Ligand-gated channels	*Grik5*	GluK5	5	5	3	5
Mechanosensitive channels	*Tmem120A*	TACAN	4	6	2	4
Pump	*Atp2b1*	PMCA1	9	/	10	9

Blue and green: genes that predominate at the onset and end of corticogenesis. Red: gene with a high expression level after the onset of corticogenesis. Yellow: major genes at all stages.

**Table 2:** List of the top 10 genes coding for intracellular Ca^2+^ transport systems.

Ca Transport System	Gene	Protein	Rank Number			
			E11	E13	E17	PN1
« Leak » pathways	*Tmem109*	TMEM109	9	/	/	/
						
« Leak » pathways	*Tmco6*	TMCO6	/	10	/	/
Transporter	*Tmem165*	TMEM165	7	9	/	/
« Leak » pathways	*Sec61b*	SEC61B	6	6	10	/
« Leak » pathways	*Panx1*	PANX1	/	/	6	6
Mitochondrial actor	*Letm1*	LETM1	/	/	9	9
« Leak » pathways	*Lrrc8b*	LRRC8B	/	/	/	10
« Leak » pathways	*Tmbim3*	TMBIM3	2	1	1	1
« Leak » pathways	*Tmbim5*	TMBIM5	4	4	5	5
« Leak » pathways	*Sec61a1*	SEC61A1	1	2	3	4
« Leak » pathways	*Sec61a2*	SEC61A2	8	8	8	8
« Leak » pathways	*Tmbim6*	TMBIM6	3	3	2	3
Channels	*Trpml1*	TRPML1	10	7	7	7
Pump	*Atp2a2*	SERCA2	5	5	4	2

Ranking is also given. / means not present in the top 10 list at a given age. The analysis encompasses a total of 14 genes. Blue and green: genes that predominate at the onset and end of corticogenesis. Pink: gene with a high expression level during corticogenesis but not after the completion of neurogenesis. Yellow: major genes at all stages.

## Supplementary Materials

Supplementary File 1
