# Supplementary material for: Transcriptomic Profiling of Ca2+ Transport Systems during the Formation of the Cerebral Cortex in Mice
Source: Cells. 2020 Jul 29;9(8):1800. doi: 10.3390/cells9081800 (PMC7465657; doi:10.3390/cells9081800)

Supplementary data

Figure S1

Neuroplastin (*nptn*) and basigin (*bsg*) are mutually exclusive auxiliary subunits of PMCA. The figure below shows the mRNA abundance (in transcripts per million, TPM) of these mandatory components of native PMCA complexes. TPM values of the *Atp2b1* gene are also given (same data as in Figure 4A).

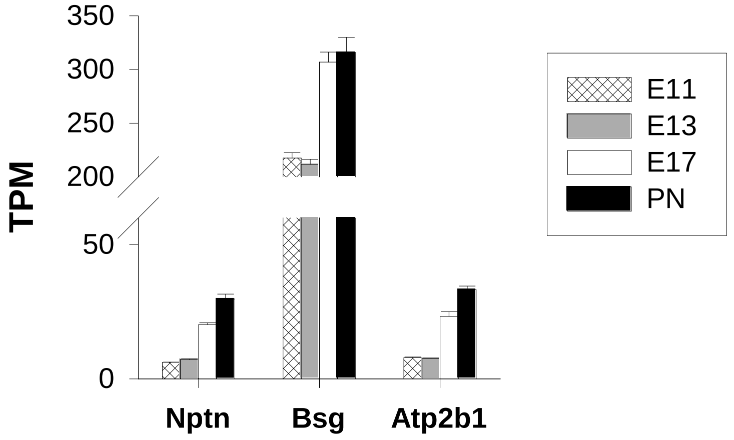

Supplement: Supplementary File 1 [file cells-09-01800-s001.pdf]
